# Prolonged polyarthralgia in a German traveller with Mayaro virus infection without inflammatory correlates

**DOI:** 10.1186/1471-2334-13-369

**Published:** 2013-08-08

**Authors:** Christian Theilacker, Jürgen Held, Ludger Allering, Petra Emmerich, Jonas Schmidt-Chanasit, Winfried V Kern, Marcus Panning

**Affiliations:** 1 Universitätsklinik Freiburg i.Br, Medizinische Klinik II / Sektion Klinische Infektiologie, Hugstetter Str. 55, 79106 Freiburg i.Br, Germany; 2 Universitätsklinik Freiburg i.Br, Department f. Medizinische Mikrobiologie und Hygiene, Institut f. Medizinische Mikrobiologie, Hermann-Herder-Str. 11, 79104 Freiburg; 3 Bernhard-Nocht-Institut f. Tropenmedizin, Bernhard-Nocht-Str 74, 20359 Hamburg, Germany; 4 Universitätsklinik Freiburg i.Br, Department f. Medizinische Mikrobiologie und Hygiene, Institut f. Virologie, Hermann-Herder-Str. 11, 79104 Freiburg

**Keywords:** Mayaro virus, Alphavirus, Persistent arthralgia, Inflammatory cytokines

## Abstract

**Background:**

Mayaro virus is endemic in South America and sporadic outbreaks have been described. It causes a dengue-like febrile illness accompanied by severe and long-lasting polyarthralgias. Outside endemic regions, however, the disease is not well known and can be misdiagnosed as dengue. International travellers are at risk to acquire Mayaro virus and due to increased worldwide travel infectious disease specialists need to be aware of such rare clinical entities.

**Case presentation:**

We report the first Mayaro virus infection imported into Germany. A 20-year-old woman developed fever, myalgia, maculopapular rash, and polyarthralgias following a 10-day trip in the Rurrenabaque region of Bolivia. Severe polyarthralgias persisted for 5 months and were treated with non-steroidal anti-inflammatory drugs. Serological analysis demonstrated Mayaro virus-specific-IgM and -IgG antibodies two months after onset of symptoms. Except for CXCL8/IL-8 other proinflammatory chemokines and cytokines were unremarkable at this time.

**Conclusions:**

Dissemination of knowledge on rare disease might improve patient management. Understanding the inherent features of Mayaro virus infection and how the virus interacts with its host are essential for optimal patient care and therapy.

## Background

Mosquito-borne alphaviruses belong to the family *Togaviridae* and have a worldwide distribution. They can be associated with rheumatic disease in humans. The most important arthritogenic alphaviruses are Barmah Forest virus (BFV), chikungunya virus (CHIKV), Ross River virus (RRV), Sindbis virus (SINV), and Mayaro virus (MAYV). Occasionally, these arthritogenic alphaviruses can cause large and unpredictable outbreaks as seen in the 2004–2011 CHIKV epidemic in the Indian Ocean [1]. Geographic range expansion of alphaviruses and increased worldwide travel are of emerging public health concern [2,3]. Of note, importation of alphaviruses into non-endemic regions can delay proper treatment as doctors are not familiar with the diseases and reliable diagnostic methods are lacking at large [3,4]. Disease in humans is usually self-limiting, but polyarthralgia can be debilitating and long-lasting. This prominent feature has been reported in particular in cases of CHIKV and MAYV infection. However, little is known about the underlying mechanism of polyarthralgia. It has been postulated that the inflammatory immune response can contribute to the pathology [5]. Here, we describe the first MAYV case imported into Germany and present data on the inflammatory immune response during the polyarthralgic stages of the disease.

## Case presentation

In July 2012, a 20-year old woman presented to our outpatient clinic with symmetrical polyarthritis after a long-term travel to South America. At presentation, she complained about highly painful ankles and elbows, and she was barely able to walk without assistance. Between September 2011 and June 2012 she had travelled as a tourist through Peru, Bolivia, and Ecuador. In Mid-April 2012 she spent 10 days in the tropical rainforest of the Rurrenabaque region, which is situated in the Amazon basin about 400 km north of La Paz, Bolivia. There, she volunteered in a wildlife resort, taking care of a variety of monkeys and wild cats. One day after leaving the resort she developed spiking fevers, headache, myalgia followed by aphthous oral ulcers and a maculopapular rash of the whole body four days later. Around the same time she developed a diffuse arthralgia, which after initial improvement slowly worsened and became highly incapacitating. Her previous medical history was unremarkable. She did not take regular medication. Prior to her travel, she had received complete vaccinations for yellow fever, rabies, hepatitis A and B.

On physical examination tenderness and mild swelling of both ankles was noted. Tenderness of the elbows, shoulders and interphalangeal joints and wrists without swelling was also observed.

Results of a full blood count, liver and renal function tests were normal, as were levels of electrolytes, lactate dehydrogenase, and C-reactive protein. Blood cultures remained sterile. Blood smears for malaria were repeatedly negative. Serologic testing for cytomegalovirus, hepatitis C virus, human immunodeficiency virus, *Borrelia burgdorferi*, and *Treponema pallidum* were negative. Serology for Epstein-Barr virus and parvovirus B19 was indicative of past infection. A slightly raised dengue virus (DENV) IgG was interpreted as cross-reactivity after yellow fever vaccination while DENV IgM was negative. In addition, autoimmune serology including anti-citrullin peptide antibodies or anti-nuclear antibodies was negative.

Due to her travel to the Amazon basin and prolonged course of arthralgia, MAYV infection was considered as a differential diagnosis. Serologic testing for mosquito-borne alphaviruses using the indirect immunofluorescence test (IIFT) and virus neutralization test (VNT) confirmed the diagnosis of MAYV infection (Table 1). Testing for MAYV RNA was not performed, since the time of presentation was more than 2 months after disease manifestation.

**Table 1:** Serological testing for alphaviruses

	**Indirect immunofluorescence assay**				**Virus neutralization assay**	
	First serum sample*		Second serum sample**		First serum sample*	Second serum sample**
**Virus**	**IgM**	**IgG**	**IgM**	**IgG**		
Mayaro virus (MAYV)	1:10240	1:160	1:20480	1:160	1:80	1:160
Semliki forest virus (SFV)	1:20	< 1:20	1:40	< 1:20	1:40	1:40
O’nyong-nyong (ONNV)	1:20	< 1:20	1:40	< 1:20	< 1:20	< 1:20
Chikungunya virus (CHIKV)	1:20	< 1:20	1:40	< 1:20	< 1:20	< 1:20
Ross river virus(RRV)	1:20	< 1:20	1:20	< 1:20	< 1:20	< 1:20
Barmah forest virus (BHV)	1:20	< 1:20	1:20	< 1:20	< 1:20	< 1:20
Sindbis virus (SINV)	1:20	< 1:20	1:20	< 1:20	< 1:20	< 1:20
Western equine encephalitis virus (WEEV)	1:20	< 1:20	1:20	< 1:20	< 1:20	< 1:20
Eastern equine encephalitis virus(EEEV)	1:20	< 1:20	1:20	< 1:20	< 1:20	< 1:20
Venezuelan equine encephalitis virus (VEEV)	1:20	< 1:20	1:20	< 1:20	< 1:20	< 1:20

*Serum sample day 83 after onset of symptoms.

**Serum sample day 106 after onset of symptoms.

Antibody titers were measured by indirect immunofluorescence test and virus neutralization test. Shown are results on day 83 and 106 after onset of symptoms, respectively.

Serum samples collected 83, 106, and 167 days after onset of symptoms, respectively, were analyzed for chemokine/cytokine levels using the Cytometric Bead Array (CBA) Human Chemokine Kit and Human Th1/Th2 Cytokine kit (Becton Dickenson, Heidelberg, Germany) according to the manufacturer’s instructions.

Except for CXCL8/IL-8, which was elevated more than 15-fold on day 83 after onset of symptoms, chemokines (CCL5/RANTES, CXCL9/MIG, CCL2/MCP-1, and CXCL10/IP-10) and cytokines (IL-2, IL-4, IL-5, IL-10, TNF-α, and IFN-γ) were not different to healthy controls (n = 5 samples) (Table 2).

**Table 2:** Analysis of cytokine/chemokine levels

	**Cytokine/chemokine level in pg/ml**			
**Cytokine/chemokine**	**Serum day 83**	**Serum day 106**	**Serum day 167**	**Control (mean)**
CXCL8/IL-8	45,9	4,1	2,7	2,9
CCL5/RANTES	10277	10218	6958	10005
CXCL9/MIG	46	31,6	20,1	22,8
CCL2/MCP-1	70,1	80,9	85,6	66,1
CXCL10/IP-10	129,6	77,2	61,4	43,3
IL-2	0	0,1	0,9	0,01
IL-4	0	0	0	0
IL-5	0,2	0,3	0,5	0,1
IL10	0,8	0,5	0,9	0,8
TNF-α	0	0	0	0
IFN-γ	0	0,5	0	0

Cytokine and chemokine response as measured on three consecutive dates within the chronic stage of MAYV disease. The control represents the mean of 5 individual determinations of 5 healthy volunteers.

The patient was treated symptomatically with non-steroidal anti-inflammatory drugs (NSAID). During follow up visits the patient reported persistent arthalgias of both ankles and elbows, which slowly improved over time. The patient continued to take NSAID for over 2 months and her symptoms finally resolved after 5 months.

## Discussion

To our knowledge, this MAYV case is the fourth of a European patient with a travel history to South America and in particular the first imported MAYV case into Germany [4,6,7]. MAYV infection is still rare in travellers and consultation of infectious disease specialists is instrumental in making the correct diagnosis [4].

Our patient presented with a travel and exposure history typical for an infection with an arthritogenic alphavirus and displayed symptoms identical to reports published previously [4,6,7]. However, knowledge about exotic alphaviruses is rather limited among general practitioners and MAYV infection might frequently be misdiagnosed as dengue on clinical grounds. In our case serologic results rapidly confirmed the clinical diagnosis supporting the importance of specific laboratory tests. It should be noted, however, that diagnostic tests for exotic alphaviruses are not widely available hampering the diagnosis in routine practice.

VNT is considered gold standard for the diagnosis of MAYV infection. However, the requirement for a biosafety level 3 laboratory to conduct these assays considerably limits their utility. IIFT and ELISA have been also described, but they are prone to cross-reactivity between alphaviruses [4,6]. Diagnosis in our case was unambiguous and no significant cross-reaction with related alphaviruses was observed. Interestingly, MAYV IgM antibodies were detectable for at least 3,5 months. It remains speculative if persisting IgM can serve as a diagnostic indicator of severe disease and arthritis [8,9].

Beyond pure case detection it should be noted that travellers can potentially act as sentinel for emerging infectious diseases [10]. In light of the spread of competent vectors for mosquito-borne diseases in Europe and elsewhere, surveillance of vector-borne diseases is of major public health importance. Therefore, the dissemination of reliable diagnostic methods is an important prerequisite both for individual and public health as experienced with other emerging infections, e.g. CHIKV and west nile virus [3,11].

Among the arthritogenic alphaviruses MAYV is restricted to South America and infections have been reported from countries including Brazil, Bolivia, Colombia, the Guiana’s, and Venezuela. Sporadic outbreaks involving up to 100 cases have been described in several regions [12-14]. Resident forrest workers and hunters are at highest risk to acquire MAYV infection [4,6,7]. In South America, MAYV is responsible for about 1% of arbovirus-associated febrile illness [15]. The virus is transmitted by bites of *Haemagogus janthinomys* which lives in the forest canopy and propagates in a sylvatic cycle predominantly in monkeys [16,17]. Hence, working and living in the Amazon rain forest is the most important risk factor for the acquisition of MAYV infection [12,13,18]. Clearly, our patient was at risk while working in a wildlife resort. Recently, however, also cases of urban transmission of MAYV infection have been described [19].

From a clinical perspective arthritogenic alphaviruses cause a similar picture consisting of acute fever, malaise, headache, maculo-papular rash, myalgia lasting for 3 – 7 days, and a characteristic and often debilitating polyarthraglia/polyarthritis lasting up to 6 months [20]. A rash is found in 20 – 60% of patients [14,19,21] In a classical fashion our patient reportedly suffered from these cardinal symptoms during the acute phase of infection. Viraemia is short-lived and controlled by the innate immune system as well as the appearance of antibodies after 5–7 days. The hallmark of all arthritogenic alphaviruses are sometimes long-lasting polyarthralgias and/or polyarthritis. There is evidence that virus induced inflammation is responsible for these arthropathies [20]. To the best of our knowledge no reports on the inflammatory cytokine response in MAYV infection are available to date. Supporting the findings of Chow et al., who analyzed patients with persistent arthralgia induced by CHIKV infection, the Th1/Th2 response remained below the limit of detection at all time points in our patient. A slightly elevated IL-8 concentration could be interpreted as waning levels after acute infection [5]. Interestingly, Chow et al. could show that levels of IL-6 and granulocyte macrophage colony-stimulating factor were associated with persistent arthralgias. Further studies on MAYV are needed, as both markers were not included in our panel. Although TNF-α and INF-γ are involved in chronic inflammatory diseases such as rheumatic arthritis and were detected in a number of arthritogenic alphavirus infections we did not detect increased levels in our patient. Differences in genetic background, disease severity or the etiologic agent may account for this finding and require further studies. Beyond the analysis of cytokines/chemokines in serum the analysis of gene expression profiles of these immune mediators may shed more light on the immune response in MAYV infection.

## Conclusions

Here, we report the first case of MAYV associated arthritis in a returning traveller from Germany. No inflammatory correlate was detectable despite long term sequelae. With increasing intercontinental travel, infectious disease specialists and rheumatologists need to be aware of alphavirus infection as a cause of febrile illness followed by persisting polyathralgia. Our clinical case illustrates that MAYV infection should be considered after travel to tropical South America, especially, if the travel itinerary included extended exposure in the Amazon rain forest and its wildlife.

### Consent

Written informed consent was obtained from the patient for publication of this case. A copy of the written consent is available for review by the Series Editor of this journal.

## Competing interests

The authors declare that they have no competing interests.

## Authors’ contributions

CT and MP drafted and wrote the manuscript. CT and WVK took care of the patient. JH and MP carried out the cytokine assays. LA, PE, and JSC carried out the IIFT and VNT. WVK critically revised the manuscript. All authors have read the manuscript and approved its final version.

## Pre-publication history

The pre-publication history for this paper can be accessed here:

http://www.biomedcentral.com/1471-2334/13/369/prepub
